# The Exploitation of Orphan Legumes for Food, Income, and Nutrition Security in Sub-Saharan Africa

**DOI:** 10.3389/fpls.2022.782140

**Published:** 2022-05-17

**Authors:** Jacob Olagbenro Popoola, Oluwadurotimi Samuel Aworunse, Omena Bernard Ojuederie, Babasola Daniel Adewale, Oluwapelumi Comfort Ajani, Olaniyi Ajewole Oyatomi, Davelyne Ifechukwude Eruemulor, Taofeek Tope Adegboyega, Olawole Odun Obembe

**Affiliations:** ^1^Department of Biological Sciences, Covenant University, Ota, Nigeria; ^2^Department of Biological Sciences, Biotechnology Unit, Kings University, Ode-Omu, Nigeria; ^3^Food Security and Safety Niche Area, Faculty of Natural and Agricultural Sciences, North-West University, Mmabatho, South Africa; ^4^Department of Crop Science and Horticulture, Federal University Oye-Ekiti, Ikole-Ekiti, Nigeria; ^5^Genetic Resources Center, International Institute of Tropical Agriculture (IITA), Ibadan, Nigeria; ^6^Biology Unit, Faculty of Science, Air Force Institute of Technology, Nigerian Air Force Base, Rafin Kura, Kaduna, Nigeria; ^7^UNESCO Chair on Plant Biotechnology, Plant Science Research Cluster, Department of Biological Sciences, Covenant University, PMB, Ota, Nigeria

**Keywords:** climate change, food security, malnutrition, orphan legumes, sustainable development goals

## Abstract

Poverty, food, and nutrition insecurity in sub-Saharan Africa (SSA) have become major concerns in recent times. The effects of climate change, drought, and unpredictable rainfall patterns threaten food production and sustainable agriculture. More so, insurgency, youth restiveness, and politico-economic instability amidst a burgeoning population requiring a sufficient and healthy diet remain front-burner issues in the region. Overdependence on only a few major staple crops is increasingly promoting the near extinction of many crops, especially orphan legumes, which possess immense potentials as protein and nutritional security crops. The major staple crops are declining in yield partly to their inability to adapt to the continuously changing climatic conditions. Remarkably, the orphan legumes are climate-smart crops with enormous agronomic features which foster sustainable livelihood. Research efforts on these crops have not attained a reasonable comparative status with most commercial crops. Though many research organizations and scientists have made efforts to promote the improvement and utilization of these orphan legumes, there is still more to be done. These legumes’ vast genetic resources and economic utility are grossly under-exploited, but their values and promising impacts are immeasurable. Given the United Nations sustainable development goals (SDGs) of zero hunger, improved nutrition, health, and sustainable agriculture, the need to introduce these crops into food systems in SSA and other poverty-prone regions of the world is now more compelling than ever. This review unveils inherent values in orphan legumes needing focus for exploitation *viz*-a-*viz* cultivation, commercialization, and social acceptance. More so, this article discusses some of the nutraceutical potentials of the orphan legumes, their global adaptability, and modern plant breeding strategies that could be deployed to develop superior phenotypes to enrich the landraces. Advanced omics technologies, speed breeding, as well as the application of genome editing techniques, could significantly enhance the genetic improvement of these useful but underutilized legumes. Efforts made in this regard and the challenges of these approaches were also discussed.

## Introduction

Africa’s population is currently estimated above 1.3 billion, and it is expected to hit 2.5 billion by the year 2050 (Fischer et al., 2009; Paliwal et al., 2021). This burgeoning figure is mounting pressure on food production. At the same time, factors such as poor soils, land degradation, climate change, lack of access to fertilizers, poor agricultural infrastructures, banditry, and insurgency impede sustainable agriculture. The continent is endowed with a rich agrobiodiversity and has excellent potential for self-sufficiency in food production. However, it is one of the food- and nutrition-deficient regions in the world. Africa’s agrobiodiversity is continuously under threat resulting in the erosion of valuable genetic resources (Popoola et al., 2020; Ikhajiagbe et al., 2021). The destruction of many agricultural fields and farms by overgrazing and unregulated nomadic pastoralism has fueled an upsurge in herder-farmer’s conflict in some regions, thereby subjecting many to poverty. Moreover, the internal displacement of persons is on the increase. Traditional agronomic practices for many indigenous species are on the verge of being lost, resulting in low yield and neglect. This could have far-reaching consequences on global food production and the supply chain. Whereas food insecurity and dietary deficiencies are global issues, their severities are more pronounced in Africa and some parts of Asia (Li and Siddique, 2020; Popoola et al., 2020). Nutritional transitions, overdependence on a few major staple foods, and improved socioeconomic status contribute to food and protein insecurity (Dixon, 2009; Chakona and Shackleton, 2017). Mitigating the impacts of these problems calls for global attention and concerted efforts by all stakeholders.

The promotion of indigenous biodiversity and incorporation of nutrient-dense crops into food value chains have been identified as measures that could ensure sustainable and resilient food systems, improve diet quality, and increase smallholder farmers’ incomes (Padulosi et al., 1999; Cheng et al., 2019; Hunter et al., 2019). The United Nations SDGs of zero hunger, achieving food security, improving nutrition, and promoting sustainable agriculture, are critical to alleviating poverty and malnutrition in Africa. To ensure sustainable food production and overcome dietary deficiencies, several strategies, including consistent cultivation, conservation, and genetic characterization of many unrecognized, abandoned, and under-exploited species alongside the reformation of traditional agronomic practices, are imperative (Popoola et al., 2020; Ikhajiagbe et al., 2021). Dietary diversification and incorporation of resilient crop species such as under-exploited leguminous species referred to as an orphan, minor, or underutilized into food systems will ensure food and protein security in many parts of Africa (Considine et al., 2017; Cullis and Kunert, 2017; Singh and Fernandes, 2018).

The term “Underutilized” or “orphan” alludes to the neglect of a species by international and indigenous research communities. Orphan species are generally classified as crops with little relevance at the global level (Muhammad et al., 2020). By way of definition, underutilized legumes are a diverse collection of domesticated pulses with beneficial properties, but with limited importance compared to major global crops like rice, maize, potato, and wheat due to utility and supply constraints. This set of crops regardless of their neglect is characteristically diverse with cultural values and inherent useful agronomic, genetic, and biochemical traits (Cullis and Kunert, 2017; Khan et al., 2021a). Orphan legumes play a vital role in several developing countries by generating revenue for smallholder farmers, as well as tackling micronutrient and protein deficiencies associated with the consumption of high calorie diet derived from the major crops researched by scientists and private corporations. Although these crops may be extensively distributed beyond their centers of origin, orphan legumes occupy special niches in traditional production and consumption systems. Whereas underutilized legumes are stapled food crops in many rural communities in sub-Saharan Africa (SSA), their economic potential for regional and international markets have not been fully tapped into, hence their neglected status and lack of genetic improvement, resulting in reduced quality and yield (Cullis and Kunert, 2017; Mabhaudhi et al., 2017). Although it is difficult to precisely define what attributes makes a crop underutilized, orphan legumes are marked by the following constraints: (i) often associated with the cultural heritage of their places of origin; (ii) poor documentation of their cultivation and use; (iii) adaptation to specific marginal land and agro-ecological niches; and (iv) no formal seed supply systems. In addition, the hard-to-cook (extended time required to achieve desired softening during cooking) characteristic, inadequate processing techniques, and seed coat hardness of many orphan legumes results in a lack of functional value chain that guarantees the delivery of processed and refined products from farmers to consumers (Khan et al., 2021c). Major legumes with a high quantity of oils particularly soybean and groundnut are preferred to orphan legumes in some regions owing to their numerous uses (Nedumaran et al., 2015; Muhammad et al., 2020). Consequently, orphan legumes which are subsistence food crops for the indigenous population are replaced with these profit crops given their export potentials (Muhammad et al., 2020). In Africa, many orphan legume crops abound which due to their dense nutritional profile, good adaptability to adverse climatic scenarios, and ability to grow in marginal soils hold potential for sustainable cultivation (Gulzar and Minnaar, 2017).

This review focuses on five orphan legume crops endemic to SSA. The crops include African yam bean (AYB; *Sphenostylis stenocarpa*), Bambara groundnut (BG; *Vigna subterranean*), Kersting’s groundnut (KG; *Macrotyloma geocarpum*), Lima bean (LB; *Phaseolus lunatus*), and Jack bean (JB; *Canavalia ensiformis*). In addition to the above-listed features, the criteria of low production rank and *per capita* consumption coupled with common trade in terms of export and import volumes (tons) at the international market compared to mainstream pulses like soybeans (*Glycin max*) and groundnut (*Arachis hypogaea*) were used in the selection of the five species. Although yield, production, and consumption of pulses had increased modestly in the last decades in SSA and on the global scale, orphan legume crops were not classed as commodities in the international trade framework. Statistics on their production, consumption, and trade remain scanty and, in some cases, nonexistent or aggregated with other pulses/dry beans. Therefore, this paper unveils inherent values in the selected orphan legumes needing focus for exploitation *viz*-a-*viz* cultivation/production, commercialization, and social acceptance. More so, this article discusses some of the nutraceutical potentials of the orphan legumes, their global adaptability, and modern plant breeding strategies that could be deployed to develop superior phenotypes to enrich the landraces. Also discussed are current biotechnology approaches and plant breeding strategies, including advanced omics technologies, speed breeding, genome editing tools, and the challenges of these approaches.

## Production and Consumption Status of Orphan Legumes

**Figure 1:**
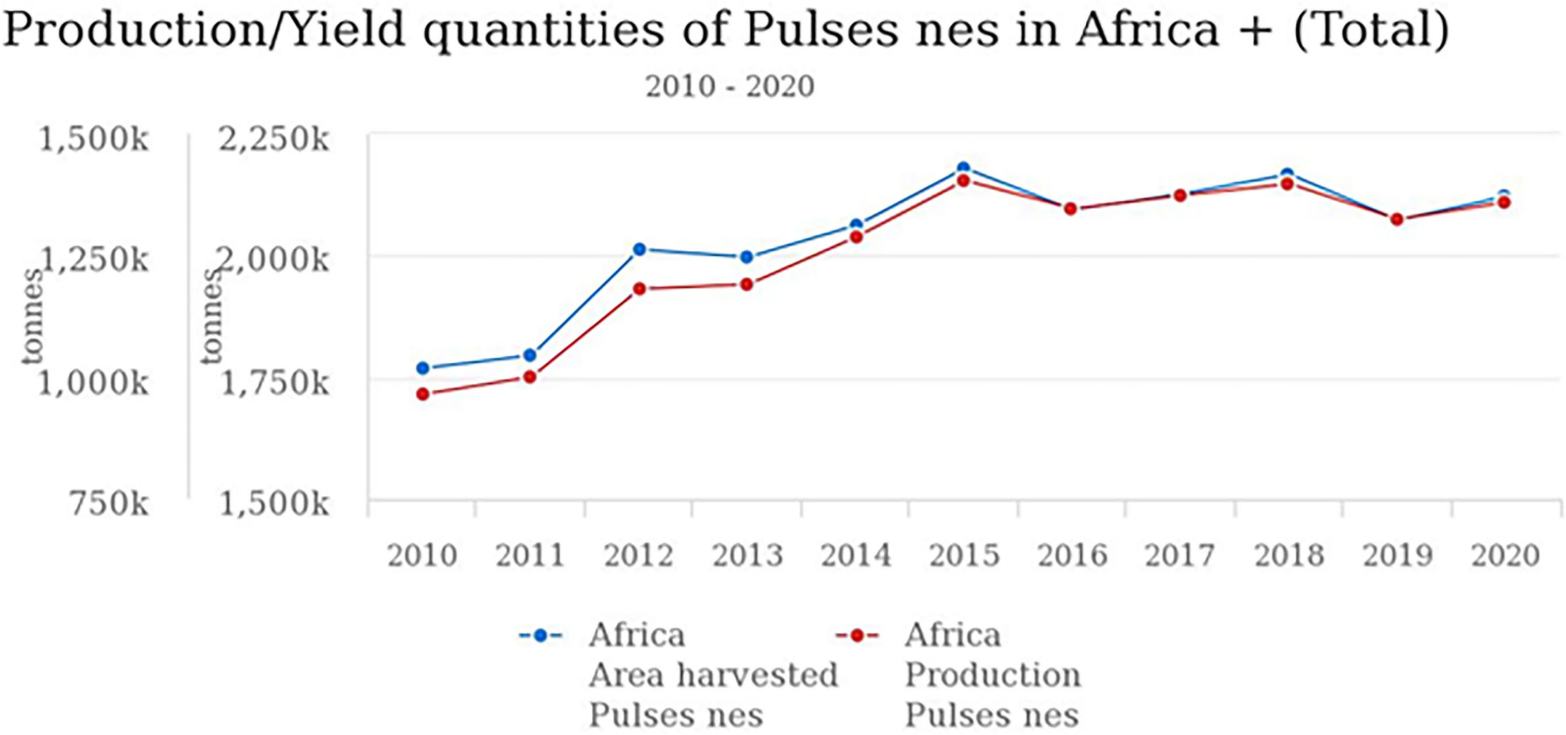
Pulses production/yield quantities in Africa.

**Figure 2:**
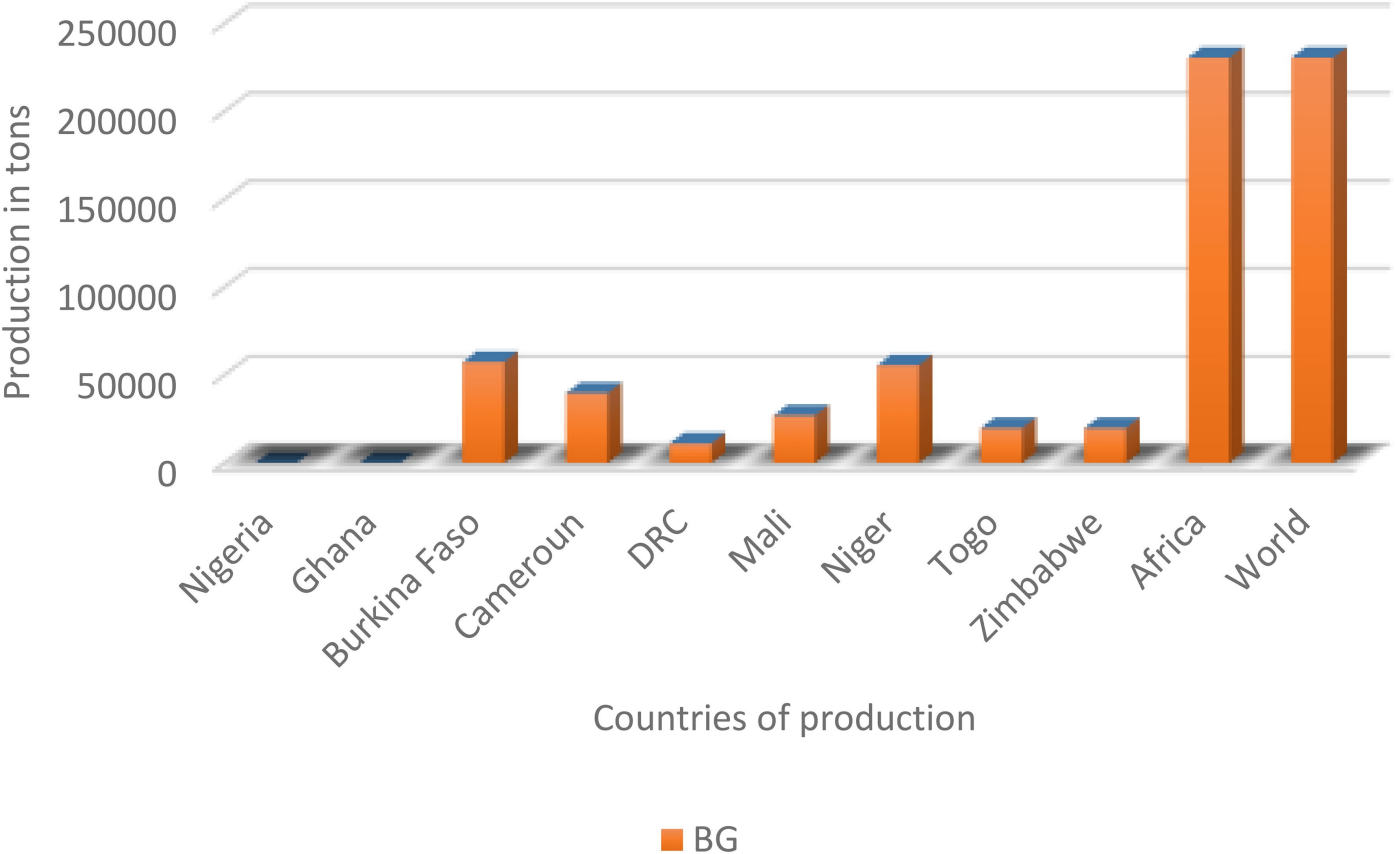
Production in tons of Bambara groundnut (BG). Only BG has data in FAOSTAT.

The production and yield quantities of pulses in Africa have surged in the last decade from 2010 to 2020, with a fall between 2015 and 2016 and a steady rise from 2017 to 2020 (Figure 1). Globally, in 2020, over 5.9 million hectares (Ha) of pulses were cultivated with a production of over 4.4 million tons (MT) and a yield of 7,503 kg/ha. Africa produced only about 1.35 MT of pulses on a harvested area of 2.12 million ha with a yield of 6,407 kg/ha. In the same year, the global production of soybean and groundnut was above 350 MT and 53 MT compared to 0.23 MT for BG (FAOSTAT, 2020). Currently, BG production is restricted to only seven countries: Burkina Faso, Cameroun, Democratic Republic of Congo (DRC), Mali, Togo, Niger, and Zimbabwe. Based on FAOSTAT, there are no production data on AYB, KG, LB, and JB (Table 1; Figure 2). Burkina Faso is the leading producer of BG with 57,429 tons, followed by Niger (55,570 tons) and Mali (26,996). Previously 100,000 tons were reported from Nigeria (Hillocks et al., 2012), but this is not captured on the current FAOSTAT data. The overview of the global area and production of pulses show that more tons of soybean and groundnut were produced globally and in SSA compared to BG and lack of production in others. The five orphan legume crops lag with little or no production data or aggregated under other pulses/beans compared to the major pulses (Ayenan and Ezin, 2016; FAOSTAT, 2020). Lima bean production is not reflecting on FAOSTAT, and just like BG, its production and that of others might not likely exceed 0.23 MT recorded for BG.

**Table 1:** Major producing countries of five selected orphan legumes of SSA and global production statistics FAOSTAT (2020).

Crop name	Botanical name	Endemic areas	Major producing countries [Production in million tons (MT)]			Total production in SSA	Global production (MT)
			1st	2nd	3rd		
African yam bean	*Sphenostylis stenocarpa*	West/East Africa	NA	NA	NA	NA	NA
Bambara groundnut	*Vigna subterranean*	West Africa	Burkina Faso (0.06)	Niger (0.056)	Cameroun (0.04)	0.23 (100% of global production)	0.23
Kersting’s groundnut	*Macrotyloma geocarpum*	West Africa	NA	NA	NA	NA	NA
Lima bean	*Phaseolus lunatus*	Tropical Africa	NA	NA	NA	NA	NA
Jack bean	*Canavalia ensiformis*	West/East Africa	NA	NA	NA	NA	NA
Soybean*	*Glycin max*		South Africa (1.45)	Nigeria (1.10)	Namibia (0.28)	1.7 (0.5% of global production)	353
Groundnut*	*Arachis hypogaea*		Tanzania (6.9)	Cameroun (5.0)	Nigeria (4.5)	16.9 (33.9% of global production)	54
Overview of global and SSA production statistics of pulses and other grain legumes, 2020.							
Legumes	Area harvested (Ha)		Production (tons)		Yield (Kg/Ha)		
	Africa	Global	Africa	Global	Africa	Global	
Pulses	2,122,050	5,918,039	1,359,666	4,440,414	6,407	7,503	
Soybean	2,550,972	126,951,517	3,438,611	353,463,735	13,480	27,842	
Groundnut	17,430,165	31,568,626	16,860,272	53,638,932	9,673	16,991	

NA, not available; *, major legumes: SSA, Sub-Saharan Africa. FAOSTAT, 2020.

Consumption of pulses had been driven by factors like taste, accessibility, ease of cooking, population growth, dietary needs, income level, and other supply chain constraints (Nedumaran et al., 2015). Given SSA sizeable consumption requirements, the region consumed 37% of global roots and tubers and only 21% of global pulses (OECD/FAO, 2020). The *per capita* consumption of pulses recommended by the Food and Agriculture Organization of the United Nations (FAO) is 30 g/day/person. Remarkably, the average *per capita* daily consumption of pulses in SSA has significantly increased from about 21 g/day/per in 1985 to about 33 g/day/person in recent time (Rawal and Navarro, 2019; Akah et al., 2021). However, the most consumed pulses are the common bean, soybean, and groundnut. The *per capita* consumption of common bean is about 10 g/day/person. In contrast, soybean consumption has increased tremendously (about 32 g/day/person) and is driven by multiple utilizations in the poultry, fishery, and edible oil industries. Contrarily, the consumption of orphan legumes is meagre, and their *per capita* daily consumption level is unknown or nonexistent compared to the mainstream pulses. Most orphan legumes are fallback crops usually consumed in lean periods when popular pulses are unavailable and expensive. Several factors such as poor agronomic features, high cost of production, intensive labor, lack of improved varieties, changes in dietary patterns, and most critically, the hard-to-cook (HTC) phenomenon hinders their production and consumption (Alexandratos and Bruinsma, 2012; Tan et al., 2020; Khan et al., 2021c).

## Farmer’s Perception and Cultivation of Orphan Legumes

The orphan legumes are mainly cultivated by subsistence farmers, who lack the financial capability to adopt high-input farming practices needed to grow major staple crops (Conti et al., 2019). In some areas, these crops are referred to as “the poor man’s food.” In Niger and Mali, these crops were essential food sources for poor farmers and their livestock during a period of drought that lasted over 20 years (Chivenge et al., 2015). The orphan legume crops are well appreciated by the traditional farmers who have spent most of their years in their hometowns, where the origin and usefulness of these grains are well understood. Farmers are aware of these crops’ capacity to fix atmospheric nitrogen into the soil, tolerate drought, provide medicine, efficiently utilize soil moisture through their deep root system, and survive harsh environmental conditions. However, significant constraints such as long cooking time, poor shelf-life, low yield, sensitivity to daylight, presence of potent anti-nutritional factors (ANTs) have plunged their utilization as farmers are less interested to invest their time and energy in their cultivation and production (Nnamani et al., 2017; Popoola et al., 2020). More so, poor market price, poor demand, lack of buyers, lack of improved varieties, and inadequate capital have hampered their production (Khan et al., 2021c). Generally, the acceptance and utilization of these underutilized grain legumes are declining owing to urbanization, change in market needs, migration, land degradation, and other earlier mentioned factors. It is believed that these factors consequently reduce the transfer of traditional and agronomical knowledge on these crops from the older to younger generation. Thus, reliable production data are scanty, except for BG (Khan et al., 2021c) and as highlighted in Table 1.

Currently, several researchers such as those at the Genetic Resources Center, International Institute of Tropical Agriculture (IITA), Nigeria; Crops for the Future Malaysia; African Orphan Crops Consortium, among others, are reappraising the need to adopt the orphan legumes as a means of boosting child development, improving public health, and bridging the nutritional gaps in the yearly nutritional cycles (Cullis et al., 2018; Vidigal et al., 2019; Paliwal et al., 2021). Very recently, Ojuederie et al. (2020) reported that well-processed seeds and tubers of AYB (one of the orphan legumes) could be included in meals to reduce protein malnutrition while improving food and nutritional security in Africa. All these are geared toward enhancing the utilization of such crops to increase their production. Thus, to obtain significant benefits from these underutilized grain legumes, there is a need for systematic cultivation, wide acceptance, increased utilization, strategic crop improvement and funding, and the creation of global and local market spaces to enhance consumption across the SSA.

**Figure 3:**
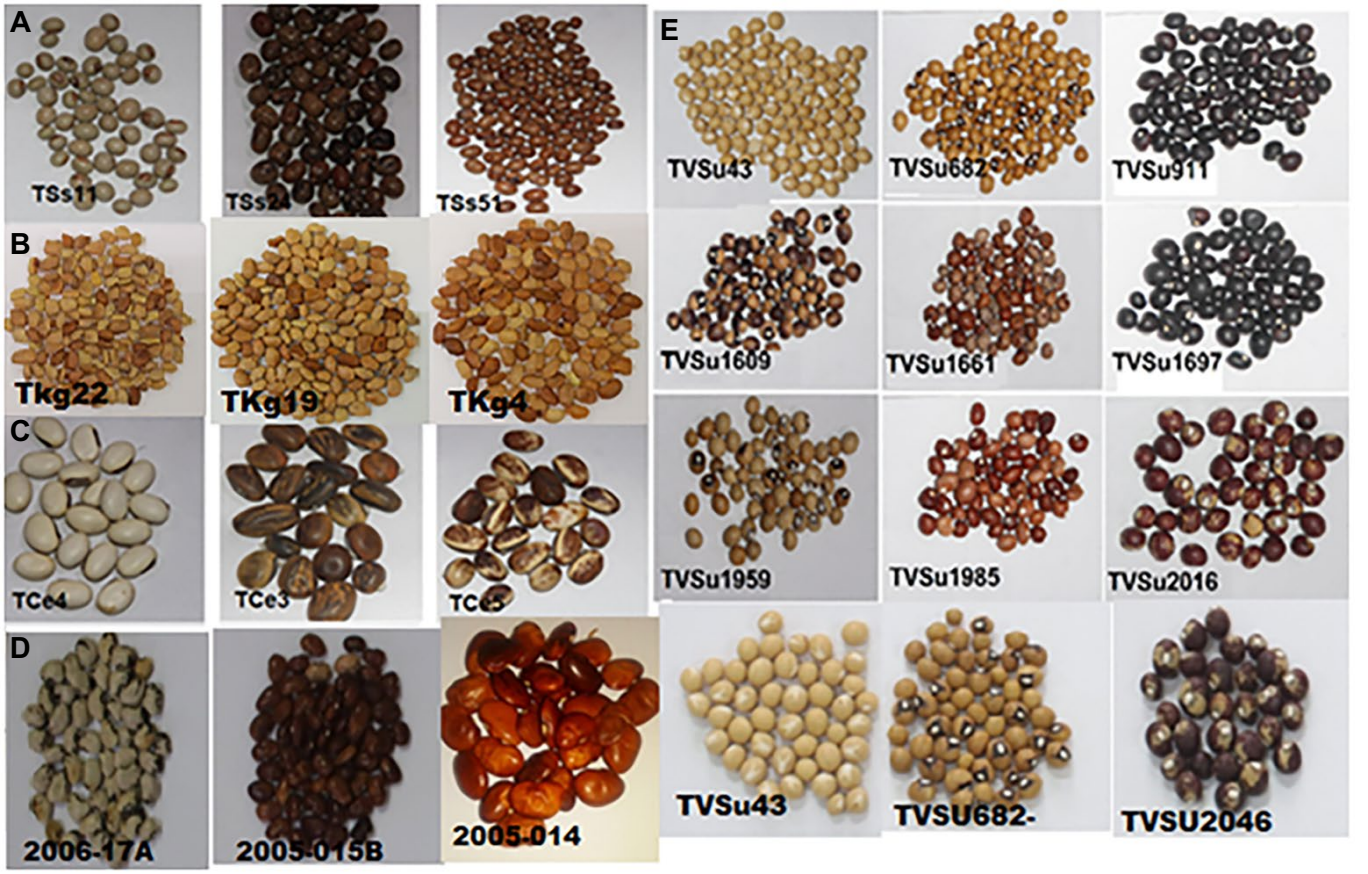
Diversity in seeds of the selected orphan legume crops. **(A)** African yam bean (*Sphenostylis stenocarpa*). **(B)** Kersting’s groundnut (*Macrotyloma geocarpum*). **(C)** Jack bean (*Canavalia ensiformis*). **(D)** Lima bean (*Phaseolus lunatus*). **(E)** Bambara groundnut (*Vigna subterranean*). Source: seeds were collected from the International Institute of Tropical Agriculture (IITA, Ibadan, Nigeria).

Diversity in seeds of the selected five underutilized legumes that can be cultivated to enhance Africa’s food and protein security is shown in Figure 3, while basic scientific information is listed in Table 2. In areas like East Africa, the Pacific, South Asia, and SSA, progress in the fight against poverty, food insecurity, and malnutrition is slow-paced compared to other regions of the world. Most importantly, the food system should be the first area of concern due to its capacity to provide nutrition to the people and stabilize the dilapidating economies of these countries. The need for the addition of food varieties like orphan legumes to the existing homogenous food-basket system in these regions can serve as safety nets to supply the nutritional needs of the people and generate support for local markets. Orphan legumes are resistant to biotic stress like pests and diseases, rich in quality protein and B vitamins, high in lysine content, and a good source of healthy oils. More so, orphan legumes are effective in fixing soil nitrogen, and capable of thriving in unfavorable environmental settings (Ojuederie et al., 2020; Popoola et al., 2020). A crop like Kersting’s groundnut is intolerant to soils with high moisture content. Cultivating this crop in drought-prone areas will have no adverse effect on the yield or nutritional content. While a few African countries such as Ethiopia, Kenya, and Malawi have emergency food reserve systems in place, some others are still unable to provide infrastructures critical to the conservation of these future food products. In the absence of appropriate storage infrastructure, embracing orphan legumes that require little or no special preservative techniques can be suitable in the short term.

**Table 2:** Basic scientific information on selected orphan legumes in SSA.

Common name	Species	Chromosome number	Basic information	References
African yam bean	*Sphenostylis stenocarpa*	2*n* = 22, 24	Rich in lysine and methionine, unlike other mainline root crops. Tubers have an appreciable amount of protein (15%) with fewer antinutrients. It can be propagated asexually by tuber and root tissue or sexually by its seed. Both the seed and tuber are edible, while the leaves are used for medicinal and forage purposes.	Adesoye and Nnadi, 2011; Popoola et al., 2011; Ojuederie and Balogun, 2019
Bambara groundnut	*Vigna subterranea*	2*n* = 22	It is a self-pollinating and fertilizing plant. Drought tolerant, resistant to high temperature, and a good source of green manure. It has a more extensive range and higher maximum concentration of crude protein than Chickpea, cowpea, and mungbean.	Halimi et al., 2019; Massawe et al., 2002
Kersting’s groundnut	*Macrotyloma geocarpum*	2*n* = 22	It is a source of cheap protein. Seeds are ground to make a local cake called “tubani.” Kersting’s seed flour, combined with maize flour at a ratio of 70–30, can be used for weaning infants. The decoction from Kersting’s leaf is used to cure venereal diseases, dysentery, and fever. Women have an essential role to play in the cultivation and marketing of this crop.	Akohoué et al., 2019; Tsamo et al., 2019
Lima bean	*Phaseolus lunatus*	2*n* = 2X = 22	It is a regular diet in Africa and is usually intercropped with cotton, coffee, maize, sweet potato, sorghum, and yam. It has both photoperiod-insensitive types and flowers in day-lengths up to 18 h, and short-length 11–13 h to initiate flower. The dry seeds are eaten boiled, fried, ground into powder and baked, and used in soups and stews. It is an excellent N-fixing legume.	Asante et al., 2008; Baudoin, 2006
Jack bean	*Canavalia ensiformis*	2*n* = 22	It is a source of food, minerals, and phytochemicals for both humans and livestock. While it is well-distributed in Africa, its nutritional, nutraceutical, and pharmaceutical benefits are largely untapped. It is also used as an ornamental plant and as a “snake repellant.”	Dada et al., 2013

## Potential of Orphan Legumes as Income Security

The global pulse trade has grown with prices more volatile and increasing faster than traded volumes (Akibode and Maredia, 2011; Nedumaran et al., 2015). This trend situates the growing global demand relative to supply. Also, the export and import of pulses had greatly expanded across SSA (Fabinin, 2021). In 2014, the international export of pulses was worth over USD 10 billion, and Africa contributed only about 9.5%, while in 2020, it grew to over USD 12 billion (ITC, 2022). Major traded pulses include soybean, groundnut, lentils, peas, chickpeas, kidney beans, and black gram. Among the five orphan legumes considered in this review, only BG is on the list and traded but of low volume. In contrast, AYB, Kersting’s groundnut, Lima bean, and Jack bean are not traded or likely bundled under other pulses/beans. The market trend for many orphan legume crops is somehow sketchy. Nevertheless, local trading and markets exist, and we predict regional and international trade expansion in the coming years as awareness increases and the value chain expands.

### African Yam Bean

This crop is grown locally in West/East/Southern African countries like Nigeria, Ghana, Benin, Cameroun, South Africa, Zambia, Uganda, and Angola. Its trade is restricted to local markets and not listed under crops and livestock products (production) of the Food and Agriculture Organization (FAO) nor listed under the Harmonized Tariff code System (HS) of the International Trade Centre (ITC). There are no data on its harvested area in hectares, production in tons, and market values. However, in Nigeria, an income of N74,000, equivalent to USD180, was generated monthly from the sales of prepared foods and derived products sold in open markets along the highways. This amount is more than twice the minimum wage (N30,000 or USUSD73) paid by the Federal Government of Nigeria (Nnamani et al., 2017). This portends a high potential to generate income for the rural population and their households.

### Bambara Groundnut

It is a pulse with a subterranean fruit set, cultivated by subsistence farmers mostly in semi-arid regions of Africa (Olanrewaju et al., 2022). BG is emerging as an income source and gradually receiving more international research attention with export potential. Currently, it is not classed as a commodity in the global trade framework due to its low production rank (Halimi et al., 2019; Hasan et al., 2021), but it is classified as an edible leguminous vegetable and falls into the Harmonized Tariff code System (HS) as fresh (HS0708) or dried (HS0713). The trade outlook for BG is promising due to its high yielding potentials in varied agro-ecological areas and available intra-Africa trade outlets. The top exporter of BG in 2020 was Madagascar, with an export value of 213,000 USD, while South Africa was the leading importer with an import value of 282,000 USD. The awareness of the potential of BG as a climate-smart crop is expanding beyond its endemic areas (Jahanshiri et al., 2022). Recently, a total of USD 6 to 13 billion of yearly income to the global economy was predicted for BG using the mean potential areas with a modesty yield of 0.85 t/ha for one optimal season (June–September) with a modest price of 143 USD/T (Mayes et al., 2019; Hussin et al., 2020; Jahanshiri et al., 2022). Currently, in Nigeria, 50 kg of BG sells for N30,000 or USD 72.30, with a considerable profit from the sales of processed products (Onuche et al., 2020). Similar information has been reported from Mali (Majola et al., 2021).

### Kersting’s Groundnut

It is a highly nutritious tropical crop adapted to drought-prone areas but neglected by researchers and policymakers. KG is declining in cultivation, and production statistics are unavailable due to intensive labor requirements, low yield, and improved varieties (Ayenan and Ezin, 2016). It is not yet traded globally, but it is well-known in Togo, Benin, Ghana, and Burkina Faso. It provides substantial incomes for the rural population, and its price can climb from CFA 1000 (USD 2) per kg in a period of plenty to CFA 4,000–5,000 (USD 8–10) per kg in a period of scarcity (Assogba et al., 2015; Akohoué et al., 2019). Farmers produce KG mainly for home consumption, and thus, market value is still low. Nonetheless, it is an essential source of income for the rural populace since gross revenue earned from growing the crop averages USD1200/ha (Assogba et al., 2015; Akohoué et al., 2019).

### Lima Bean

This crop is also known as butter beans. Though there is no matching HS code and trade statistics for LB, its trade is increasing globally and possibly lumped under the HS code for other pulses. Its utilization is expanding in the United States, Brazil, and India. Production and trade volume is low in SSA. In 2020, Morocco was the top exporter of LB with an export value of USD 271 M, while the United States was the leading importer valued at USD 198 M (Tridge Statistics, 2022)—data based on HS code 070820 (*Phaseolus spp*).^1^

### Jack Bean

This crop is grown in the traditional farming system in SSA, and there are no statistics on its trade. It has impressive nutritional contents, but it is largely untapped.

In summary, the prospects of these underutilized legumes look promising in SSA if stakeholders, including farmers, policymakers, government, and consumers, can embrace an integrated approach involving improved varieties, modern agronomic practices, increased funding, genetic manipulation using their rich genomic resources, and infrastructural development.

## Agronomic Resources of Orphan Legumes for Sustainable Livelihood in Sub-Sahara Africa

Several agronomic features confer on orphan legumes significant potential to enhance sustainable agriculture and human livelihood (Table 3). Poor research interest in these crops informs that there is no robust, comparable, and reliable empirical information which can be used to advocate for policy development (Mabhaudhi et al., 2019). This further establishes their poor competition with notable crops. However, the few reports considered in this review informed that orphan legumes are not useless but host huge possibilities for food and protein security. Critically, the continued existence of orphan crops within marginalized farming systems shows that they are adaptable to changing climatic scenarios. According to Vidigal et al. (2019), pulse crops offer a viable and sustainable strategy for upholding farming systems’ intercropping and production indices. The capacity to augment soil nitrogen makes orphan legumes an excellent partner in many farming systems, with additional contributions leading to soil fertility improvement, biodiversity conservation, ecosystem stabilization, farming risks reduction, and sustainable yield (Table 4).

**Table 3:** Exploitable agronomic wealth offered by underutilized legumes.

S/N	Details	References
1	When legumes serve as cover crops, they prevent excessive moisture loss from the soil, protect the soil from excessive heat, and conserve soil biodiversity.	Gepts et al., 2005; Adeleke and Haruna, 2012
2	Legumes leaves and fodder decomposes when returned to the soil and add organic matter and nutrients to enrich the soil and consequently boost crop growth and yields.	Gepts et al., 2005
3	Orphan legumes thrive in very harsh weather, acidic, infertile, and unsuitable soils, they are drought tolerant and enrich soil nitrogen by fixing atmospheric nitrogen.	Babalola et al., 2017
4	Intercropping with legumes improves soil fertility in poor farmlands through gradual natural amelioration, enhances land productivity, reduces pests and diseases population, and reduces yield loss to pests and diseases.	Belel et al., 2014; Dahmardeh et al., 2010
5	Soil fertility improvement through nitrogen fixation is by symbiotic activities of legumes. Legumes contribute over 45 million tons of fixed nitrogen to crop agriculture annually.	Belel et al., 2014
6	Legumes improve cation exchange capacity (CEC) in fields compared to lands where nonlegumes were previously cropped. CEC improvement in soils is usually due to the dropping and decomposition of legume leaf litters. This equally led to a decrease in soil nutrient losses.	Allito et al., 2015
7	Legumes improve soil structure, increase organic matter, improve water efficiency, save water for subsequent cropping systems, provide soil coverage, minimize soil evaporation, controls erosion and weed, etc.	Vidigal et al., 2019
8	The involvement of orphan leguminous crops provides opportunities for improvement of nutrient cycling and increase in the presence and population of pollinators, thereby protecting biodiversity and the ecosystems.	Chapin et al., 2000; FAO, 2010

**Table 4:** Agronomic Utilities, Challenges, and plausible solutions of the five orphan legumes.

Orphan legume species	Specific utilities	Specific challenges	Possible solutions	References
African yam bean (AYB; *Sphenostylis stenocarpa*)	It promiscuously and profusely nodulates. Notable symbionts are *Rhizobium* spp. and *Bradyrhizobium* spp. A very good companion crop to yam. Thrives and survives in most marginal agro-ecologies. Low cholesterol content, making it a suitable source of food for diabetic, obese, and hypertensive patients	Autogamous nature, intra-specific incompatibility often reduces hybridization successes. Low yields Indeterminate growth pattern and long gestation period. Photoperiodic sensitivity Hard-to-cook (HTC) Presence of antinutrient factors (ANFs) Mostly an obligate twiner, significant higher grain productivity is dependent on the use of stakes. Physiology/taxonomic of tuber or none tuber production of the species still need clarity Poor awareness of its nutritional potential	Biotechnology using marker-assisted crop improvement strategies, embryo rescue, genetic modification for novel trait integration, gene editing tools to overcome the HTC and ANFs. High throughput genomic technology could clear the air on the species physiology and taxonomy. Mutation breeding Extension research and activities will improve its awareness and utilization	Assefa and Kleiner, 1997; Okpara and Omaliko, 1997; Adewale and Adegbite, 2018; Baiyeri et al., 2018; Oluwole et al., 2021a
Bambara groundnut (BG; *Vigna subterranean*)	The crop can fix up to100 kg/ha of nitrogen. The rhizosphere of BG hosts nitrogen and phosphorus connections. It features different cropping systems. It is a significant companion crop in many crop rotation systems in SSA. Significantly, it is an excellent companion of sorghum, maize, yam, pearl millet in the field.	Small-sized flowers ANFs Hard-to-cook trait Hard-to-mill Photoperiodic sensitivity Pest and Diseases Lack of modern processing technology and storage Poor awareness of its nutritional potential	Crop improvement efforts, such as hybridization, mutation breeding, Targeting Induced Local Lesions in Genomes (TILLING) Resistance breeding for pest and disease control. Dehulling, boiling, fermentation, soaking, infrared heating, autoclaving are ways to overcome ANF and HTC problems. Extension research and activities will improve its awareness and utilization	Mohale et al., 2014; Mubaiwa et al., 2017, 2018; Khan et al., 2020, 2021b; Tan et al., 2020; Majola et al., 2021
Kersting’s groundnut (KG; *Macrotyloma geocarpum*) Formerly, *Kestingiella geocarpa*	Improvement of soil fertility through nitrogen fixation (16.5–57.8 kg ha^−1^) of atmospheric Nitrogen. Highly preferred meal due to its palatable taste, compared to other legumes. A rich meal with medicinal value	Displaced by *Arachis hypogea*, almost extinct in Africa Awareness still declining Unavailability of improved varieties Low yield ANFs Cultivation confinement to only the West African regions	Saving the extant germplasm in the few farmers’ hands seems the most urgent task. Its notable Rhizobium strain is Bradyrhizobium CB 756. Its exploitation can enhance land-use sustainability. The promotion of value chains to reduce the declining trend in its cultivation	Adu-Gyamfi et al., 2011; Ayenan and Ezin, 2016
Lima bean (LB; *Phaseolus lunatus*)	LB is highly nutritious and has been linked to several potential health benefits. It is a fat-free proteinous crop. Eating these protein-packed legumes may even lead to healthy weight loss, enhanced blood sugar control, and improved heart health.	Poor and declining awareness due to urbanization and agricultural and land-use practices Unavailability of improved varieties The long gestation period An obligate climber, rarely grown as a companion crop Low yield ANFs	Germplasm rescue and conservation are key to reducing the dwindling genetic resources of the crop. Diversity and crop improvement research should focus on the listed constraints to enhance utilization and awareness. Extension programs should bring up the nutritional values of the crop for increased consumption.	Baudoin, 2006.
Jack bean (*Canavalia ensiformis*)	Big-sized bean that is very important in animal and human nutrition. The protein content is between 23% and 34%. It is adequate in most essential amino acids. Carbohydrate is as high as 55%. It is a rich source of Ca, Zn, P, Mg, and Cu.	It is not very popular. Its usefulness is better known in the science domain, it mostly exists in the wild as an uncultivated legume. Its consumption by a human is rare in West Africa. Anti-nutritional factors are identified in the crop.	Germplasm rescue, conservation, and increased awareness through extension programs come first in proffering solutions to the numerous constraints of the crop. Focused research is needed on the genetic diversity of landraces and wild types to identify promising lines for high protein and nutritional quality to meet livestock, human and industrial needs.	Akpapunam and Sefa-Dedeh, 1997; Kanetro et al., 2021

### Specific Agronomic Utilities, Challenges, and Plausible Solutions of Orphan Legumes

The survival of orphan legumes in a marginal environment may be due to their capacity to stimulate the colonization of a variety of nitrogen-fixing bacteria in different soil types (Naluwairo, 2011; Baldermann et al., 2016; Mabhaudhi et al., 2019).

As documented in Babalola et al. (2017), *Allorhizobium*, *Azorhizobium*, *Bradyrhizobium*, *Mesorhizobium*, *Rhizobium*, and *Sinorhizobium* are among the nitrogen-fixing bacteria which exist naturally in legumes. Moreover, the diversity of host legumes is accompanied by diversity in nodulating bacteria with significant differential capacity at taxa and cultivar levels (Sprent et al., 2010). This seems to inform that the use of legumes in soil amelioration would be a backbone for organic agriculture if their resources are well harnessed. There are very wide variations in nodule productivity and effectivity of both plant and rhizobial germplasms for optimizing nitrogen fixation (Sprent et al., 2010). Babalola et al. (2017) hinted that there are so many underutilized leguminous crops whose potentials have not been fully tapped to understand their functionalities within the realm of biological N fixation (BNF).

Agronomic features, specific challenges, and possible solutions of the considered orphan legumes are provided in Table 4. Notably, the contribution of the nitrogen-fixing bacteria which cooperatively function with orphan legumes is not well documented. However, the reasons for the protracted featuring of the most orphan leguminous crop in the cropping systems in SSA may be due to their contribution to soil fertility which farmers may have found to be highly sustainable. We speculate that the quantity of nitrogen they supply to the soil may be very significant, although this needs to be investigated.

## Orphan Legumes and Human Dietary Deficiencies

Human dietary deficiencies are caused by a lack of essential nutrients in diets or the body’s inability to absorb and process these nutrients once ingested. Deficiency in nutritional requirements is the leading cause of various diseases today, like diabetes, protein-energy malnutrition (PEM), ‘beriberi’ (Thiamine deficiency), among others (Wokes et al., 1955; Flyman and Afolayan, 2006). Generally, legumes are a good source of quality proteins, dietary fiber, vital amino acids, and minerals, but scientific reports have shown that under-exploited legumes are nutritionally superior to the other commonly known legumes (Considine et al., 2017; Adegboyega et al., 2020). Many diseases are associated with a low diet, high in animal-based nutrients, overly processed foods, and low plant-based nutrients. Underutilized legumes are known to be a rich source of bioactive compounds, unsaturated fat, little or no cholesterol, and dietary fiber (Bhartiya et al., 2015; Li and Siddique, 2020). These dietary constituents promote health and sustainability by decreasing insulin production and preventing chronic diseases such as cancer, cardiovascular diseases, obesity, and the likes (Bhartiya et al., 2015; Salvi and Katewa, 2016; Hunter et al., 2019). A legume-based diet can guarantee a longer and healthier life. Without any doubt, the inclusion of under-exploited legumes into the dietary cycle of countries suffering from chronic deficiencies, malnutrition, hunger, and protein deficiency, will be a long-lasting solution due to its dense nutrient content, good quality protein, and micronutrients. It could also reduce the over-dependence on other major legumes like soybean, cowpea, chicken pea, groundnut, etc. In obesity, several studies have shown that consumption of processed underutilized legumes such as AYB and Lima bean could significantly aid weight loss (Onwuka et al., 2009; Crujeiras et al., 2010; Rebello et al., 2014). This could be attributed to the low-fat content and high dietary fiber. Many vegetarians include underutilized legumes as an alternative source of animal protein/meat without lagging in nutritional quality (Crujeiras et al., 2010; Vidigal et al., 2019). The low gastrointestinal tract nature of legumes’ carbohydrates aids in stabilizing the blood glucose level (Maphosa and Jideani, 2017). Generally, the B-Group vitamins such as folate, niacin, thiamin, riboflavin, pyridoxine, pantothenic acid among others are abundant in the orphan legumes (Table 5). These species are also a source of essential minerals like zinc, iron, calcium, potassium, copper, and selenium (Table 5). Calcium plays an important role in bone health and also eases the movement of blood within vessels. Zinc helps boost the immune system, iron synthesizes hemoglobin, and potassium helps prevent stroke (Barman et al., 2018). Orphan legumes with high zinc content would be very useful in preventing the symptoms associated with the Covid 19 virus if introduced into the human diet in the required amount. The high-quality protein (from 19.4 g per 100 g in KG to 22.5 g per 100 g in AYB) available in these orphan legumes makes them suitable and essential for every age group. However, some species like AYB, BG, KG, and JB are known to contain some toxic phytochemicals that can lead to bloating, flatulence, or activate allergic reactions in some people. Fortunately, most of these toxins can be easily neutralized by steaming, de-hulling, boiling, fermenting, roasting, and using advanced processing technology such as irradiation, infrared heating (micronization), and high-pressure cooking without jeopardizing their nutritional contents (Maphosa and Jideani, 2017; Adeleye et al., 2020; Tan et al., 2020; Khan et al., 2021c). Comparative nutritional profile of raw, mature seeds, and values per 100 g of the considered orphan legumes are shown in Table 5. The dense network of adequate quality protein, novel minerals, and vitamins all make up for their nutraceutical and pharmaceutical impact on human health, not exempting the animals’ health that feed on them.

**Table 5:** Nutritional profile of raw, mature seeds, and values per 100 g of five orphan legume crops.

Nutrient contents	AYB	BG	KG	LB	JB
Protein (g)	22.46^a^	18.8^e^	19.4^g^	21.46^g^	20.50^j^
Carbohydrate (g)	53.68^a^	61.30^e^	66.60^g^	63.38^g^	61.60^j^
Moisture (g)	9.53^a^	2.10^e^	1.70^h^	Nr	8.30^j^
Ash (g)	4.28^a^	2.40^e^	3.20^h^	Nr	3.00^j^
Fat (g)	3.59^a^	6.20^e^	1.10^g^^,^^i^	0.69^g^	4.10^j^
Total Dietary Fiber	7.30^b^	5.50^e^	5.50^g^	19.00^g^	7.00^j^
Water (g)	61.50^b^	10.30^e^	Nr	Nr	10.80^j^
Energy (kcal/100 g)	333.67^d^	367.00^e^	348.00^g^	338.00^g^	356.00^j^
Folates (B9; mg/100 g)	0.10^b^	0.25^f^	Nr	0.40^g^	Nr
Thiamine (B1; mg/100 g)	0.19^c^	0.61^f^	0.76^g^	0.51^g^	0.29^j^
Niacin (B3; mg/100 g)	0.07^c^	1.80^f^	2.30^g^	1.54^g^	1.40^j^
Riboflavin (B2; mg/100 g)	0.20^c^	0.31^f^	0.19^g^	0.20^g^	0.02^j^
Vitamin B6 (mg/100 g)	0.10^e^	0.44^f^	Nr	0.51^g^	Nr
Vitamin A (mg/100 g)	Nr	Nr	Nr	Nr	Nr
Vitamin C (mg/100 g)	12.97^c^	0.27^f^	0 IU^g^	0.00^g^	1.00^j^
Vitamin D (mg/100)	0.00^b^	3.42^f^	Nr	0 IU^g^	Nr
Vitamin E (mg/100 g)	0.19^b^	Nr	Nr	0.72^g^	Nr
Vitamin K (mg/100 g)	Nr	0.001^f^	Nr	0.006^g^	Nr
Pantothenic acid (B5; mg/100 g)	Nr	1.80^f^	Nr	1.36^g^	Nr
Sodium	1.00^b^	3.60^f^	5.67^h^^,^^i^	18.00^g^	18.00
Calcium (mg/100 g)	15.00^b^	1.60^f^	103.00^g^	81.00^g^	150.00^j^
Copper (mg/100 g)	0.29^b^	0.09^f^	0.20^h^	0.0007^g^	0.73^j^
Iron (mg/100 g)	1.50^b^	5.52^f^	15.00^g^	7.51^g^	6.20^j^
Magnesium (mg/100 g)	69.00^b^	7.58^f^	62.40^h^	224.00^g^	11.98^k^
Manganese (mg/100 g)	3.35^b^	0.26^f^	1.30^h^	1.67^g^	Nr
Phosphorus (mg/100 g)	99.00^b^	32.50^e^	392.00^g^	385.00^g^	272.00^j^
Potassium (mg/100 g)	419.00^b^	183.00^f^	332.00^g^	1724.00^g^	301.80^j^
Zinc (mg/100 g)	0.78^b^	0.27^f^	6.50^h^	2.83^g^	2.80^j^
Selenium (mg/100 g)	150.00^b^	Nr	Nr	0.007^g^	Nr
Beta-carotin (μg)	7.00^b^	0.47^f^	Nr	Nr	Nr

AYB, Africa yam bean; BG, Bambara groundnut; KG, Kersting’s groundnut; LB, Lima bean; JB, Jack bean and Nr, not reported.

a Baiyeri et al., 2018.

b Charrondière et al., 2020.

c Nnamani et al., 2018.

d Ojuederie and Balogun, 2017.

e Damfami and Namo, 2020.

f Adeleke et al., 2018.

g USDA Food Data Central.

h Aremu et al., 2006.

i Ayenan and Ezin, 2016.

j Indonesian Food Composition Table (IFCT), 2019.

k Ibeto et al., 2019.

Regional differences exist in the utilization of these rich protein and nutrition security crops. For instance, for the AYB, the tubers are highly consumed in east and central Africa while the seeds are consumed in West Africa. Thus, there is the tendency of having more seed production of AYB in West Africa and the focus on tuber production in the east and central Africa. Studies by (Ojuederie and Balogun, 2017) and Ojuederie et al. (2020) showed that the tubers of AYB have a high protein content of up to 15% which is considerably higher than the protein content found in tuber crops such as cassava and sweet potato. Findings of their study on Wistar rats also found the tuber to possess fewer antinutrients than the seeds. This study has led to more research interest on the tuberization of AYB for food and nutrition security. There is therefore an urgent need to increase the production of these orphan legumes and others identified as being rich in protein and essential minerals to lessen malnutrition and enhance food and nutrition security in Africa.

## Nutraceutical and Pharmacological Properties of Orphan Legumes

A nutraceutical can be defined as a food or part of a food that provides medical or health benefits, including preventing or treating a disease. It can be an isolated nutrient, dietary products, processed foods, or beverages. Legume seeds can be considered a potent nutraceutical. It benefits human health and prevents or treats several diet-related diseases such as obesity, cardiovascular diseases, digestive tract diseases, diabetes, and others (Morris, 2003; Duranti, 2006). Regardless of the dense protein content in legume seeds, they are also known to contain an adequate amount of energy, carbohydrates, minerals, vitamins, dietary fiber, and low-fat content (Ojuederie and Balogun, 2017; Adegboyega et al., 2020; Ojuederie et al., 2020). Some bioactive compounds like glycosides, tannins, isoflavones, saponins, flavonoids, and so on contribute to its nutraceutical abilities (Carbonaro et al., 2015; Hoste et al., 2015). In most extensively pigmented legume seeds such as Lima bean, Jack, and Sword beans, there is a high phenolic acid and flavonoid level, contributing to the coloring alongside anti-oxidative functions (Morris, 2003; Carbonaro et al., 2015; Serventi and Dsouza, 2020). The seeds also contain enzyme inhibitors α-amylase, α-glucosidase, and γ-aminobutyric acid (GABA), for which it can be used as a nutraceutical molecule (Barman et al., 2018). Green legume seeds are also a good source of nutraceutical compounds. When legume seeds are processed, they enhance the present nutraceuticals’ bioavailability by inactivating ANTs like trypsin, growth inhibitors, and hemagglutinins (Suneja et al., 2011; Serventi and Dsouza, 2020). Some molecules in legume seeds that have been tagged toxic can be crucial to human health if consumed in the right proportion.

Underutilized legumes can be used as active components of drugs and other pharmaceutical products to promote human health status in terms of pharmaceuticals. Most under-exploited legumes are used in folk medicine in various parts of the world without knowing their active ingredients. For example, Kersting’s groundnut can be used to cure diarrhea, BG provides relief from menstrual cramps, cures insomnia, and promotes red blood cell production as well as prevent cancer (Ayenan and Ezin, 2016; Tsamo et al., 2019; Udeh et al., 2020). Similar properties abound in most of the underutilized legumes, including the AYB seeds where the paste made from the ground seeds have been used to treat ailments such as stomach aches and acute drunkenness when mixed with water in some West African countries; Ghana, Nigeria, and Togo (Adewale and Dumet, 2010). These pharmaceutical capabilities are attributed to bioactive agents like phenols capable of signaling structural polymers such as anthocyanin, flavonoids, tannins, and phenolic acids that chelate metal ions, inhibiting the peroxidation lipids and scavenge free radicals (Morris, 2003; Serventi and Dsouza, 2020). Phenolic compounds are responsible for the anti-oxidative nature of the seeds (Serventi and Dsouza, 2020). Also, phenolic compounds show anti-bacterial, anti-viral, anti-inflammatory, anti-mutagenic activities, and cancer prevention (Morris, 2003; Serventi and Dsouza, 2020; Udeh et al., 2020). Precisely, legumes exhibit pharmacological properties such as antioxidant, antimicrobial, anticancer, anti-inflammatory, anti-obesity, and heart protection (Serventi and Dsouza, 2020). The seeds have to be fermented to get the maximum anti-oxidative potentials of phenolic compounds (Rezende et al., 2018). Triterpenoids are commonly found saponins in legumes which play a major role in reducing carcinogenic substances in the colon. They can lower the risk of heart diseases and serve as immune stimulants by inducing cytokine production such as interferon (IFN) and interleukins (Singh et al., 2017; Serventi and Dsouza, 2020). Tannins are also abundant among the orphan legumes, which helps remove toxins from the intestinal tract because of their ability to bind proteins (Hoste et al., 2015; Barman et al., 2018). Anthocyanin, alkaloids, enzyme inhibitors such as α-Amylase and α-Glucosidase, phytic acid, and phytoestrogen have been isolated and reported among the orphan legumes (Barman et al., 2018; Serventi and Dsouza, 2020). The nutraceutical components of these grain legumes are mostly used as preventive measures of disease or to reduce some infections’ virulence (Duranti, 2006; Hoste et al., 2015; Barman et al., 2018). The introduction of these under-exploited legumes into our diet at a healthy amount will be of great health benefits to people in SSA.

## Global Adaptability and Genetic Resources of Orphan Legumes

Globally, tropical Africa and some regions in Asia are the most significant endemic areas and producers of orphan legumes (Cullis and Kunert, 2017; Popoola et al., 2020; Paliwal et al., 2021). These species’ ability to thrive in various environments, whether harsh or favorable, has enabled the species to survive in diverse areas. For instance, the Lima bean (*Phaseolus lunatus*) was initially endemic to West Africa, but it is now cultivated mainly in the temperate regions of the United States (Caicedo et al., 1999; Serrano-Serrano et al., 2010; Andueza-Noh et al., 2013). Furthermore, adaptability is the capacity to acclimatize to a wide range of environmental conditions effectively. Orphan legumes should be a reference masterpiece in terms of adaptability due to their ability to overcome neglect, near extinction, and under-exploitation before becoming the highlight of scientific research. These unpleasant conditions triggered various adaptation mechanisms (survival strategies) in their morphology, physiology, biochemical, and genetic constituents (Sita et al., 2017). Such survival mechanisms include; possession of a deep taproot system to enhance soil water capture during drought, regulation of stomata, reduction of canopy size and duration, increasing wax accumulation on the surface to prevent water loss, early maturation to allow reproduction before environmental conditions depreciate (mostly in annual crops), and nitrogen-fixing ability to self-enrich their soil (Sita et al., 2017; Kumari et al., 2020). The *Canavalia* species (*C. ensiformis* and *C. gratilis*) are characterized by an extensive rooting system that can be exploited for phytoremediation of polluted sites. Generally, grain legumes have various adaptation mechanisms for different threats and stresses; this ability makes them unique, and the need for their exploitation is crucial (Considine et al., 2017; Cullis and Kunert, 2017).

Plant genetic resources (PGR) are the plant’s vegetative and reproductive parts, from which the plants are propagated. In orphan legumes, every part is of great importance, ranging from the root, seeds, tubers, pods, flowers, and stems. Each part possesses heritable characteristics of potential value to plant breeders, varying from their dietary, medicinal, and social value. Due to the effortless adaptability of orphan legumes to almost all climatic conditions, each species has thousands of accessions, varying in phytochemical content, proximate, nutritional, and antimicrobial values. The availability of a diverse collection of PGR is the building block for any crop improvement program. Future development of improved varieties of orphan legumes depends largely on collections and preservation of their PGR. The Genetic Resources Center (GRC) of the International Institute of Tropical Agriculture (IITA) presently maintains a collection of about 6,747 accessions of various orphan legumes consisting of about 2000 accessions of BG (Atoyebi et al., 2017), with 19% of these collected from Nigeria, 456 accessions of AYB with 97% from Nigeria while 100% of the 22 accessions of the Kersting’s groundnut, were collected from Nigeria. Other legumes conserved at the Genetic Resources Center of IITA include winged bean (50 accessions) and other minor legumes, such as Jack bean, Mung bean, *Canavalia species*, and rice bean, made up of about 18% of the total 25,000 accessions of the seed crops conserved at the center.

The IITA Genebank has been developing a program of research on various aspects of these orphan legumes, particularly BG, AYB, and winged bean, and lately Kersting groundnut to understand their genetic diversity, nutrient composition and to evaluate them for various biotic and abiotic stresses (Paliwal et al., 2021). To identify valuable traits for improving and developing climate-smart varieties, it becomes necessary to conduct genetic diversity studies and quantitative trait loci (QTL) discovery for drought, yield-related traits, and climate-adaptive traits. Accessions of AYB have been evaluated for both nutritional and anti-nutritional properties, the result indicated a good nutritional profile of the seeds with high protein, carbohydrate, and other nutrients when compared with other important legumes (Adegboyega et al., 2020), while the assessment of the genetic variation of AYB accessions using the established crop descriptors and molecular markers has been carried out for some of the collections (Moyib et al., 2008; Adewale et al., 2014; Ojuederie et al., 2014; Shitta et al., 2016; Nnamani et al., 2019). Studies are ongoing on the seed processing procedure that will enhance the seed’s longevity in storage for both BG and AYB to minimize the high cost of conservation of their germplasm, thereby securing the availability of their PGR for future genetic improvement. The seeds of these crops were collected and stored in genetic resource centers, while the whole plant can be propagated *in vitro* using tissue culture techniques to ensure their sustainability (Ogunsola et al., 2016).

## Global Research Efforts and Prospects

### Biotechnological Approaches to the Improvement of Orphan Legumes

Major bottlenecks like the “hard-to-cook” factor, low yield, indeterminate growth habit, pest and disease attack, pod shattering, mandatory need for stakes or trellis, low seed supply, among others, cannot be solved at once. However, plant breeders and researchers working on the orphan legumes are to tackle these issues accordingly to improve the species for sustainable utilization. Plant breeders are currently studying how the entire genome sequence of some species such as AYB and BG can hasten their genetic improvement and, hence, better utilization. The genomes of some of these legumes are currently being sequenced by the African Orphan Crops Consortium (AOCC), which seeks to assemble and annotate the genomes of 101 traditional African food crops to improve their nutritional content (Ojuederie et al., 2021). Regardless of the ongoing efforts to see to the complete success of orphan legumes in the global food basket, the need for more crop improvement strategies is demanding, not just from conventional plant breeders alone, but biochemists, plant geneticists, botanists, technology providers, the government, and the traditional farmers. They have in-depth knowledge about these minor legumes. Presently, the impact of morphological markers in analyzing the diversity among germplasms of various under-exploited legumes is minimal since environmental conditions influence results. Future directions lie in the use of genome-editing tools, e.g., the Clustered Regularly Interspaced Short Palindromic Repeats (CRISPR), GBS (Genotyping by sequencing), High-throughput phenotyping, SNP (Single nucleotide polymorphism), CNV (Copy number variation), cloning, transformation, and core set development, to get improved traits. Exploring the genomics of these underutilized crops *via* conventional breeding, Genome-Wide Association Studies (GWAS), modern biotechnology, and computational technology, will hasten the species’ improvement for wide acceptability and utilization (Paliwal et al., 2021). In recent years, the development of large-scale genomic and genetic resources, including simple sequence repeat, expressed sequence tags, diversity array technology markers (DArT), and draft genomes, have enhanced genetic knowledge on these minor species (Varshney et al., 2009; Chang et al., 2019). These discoveries can accelerate gene discovery and pioneer breeding at the molecular level in these under-exploited crops.

### Molecular Markers of Orphan Legumes

The conventional method of producing plants with improved traits relied on time-consuming and laborious approaches. It could take up to 8–12 generations to obtain an improved crop with the desired trait, but other genes could also be transferred in the process. Thus, the use of an alternative method such as modern biotechnology has been considered by several researchers for the improvement of orphan legumes. However, most of the studies that have been conducted were based on the use of DNA or molecular markers for genetic diversity studies and marker-assisted breeding of these underutilized species. Molecular markers are segments of DNA revealing variations, which can be used to detect polymorphism between different genotypes or alleles of a gene for a particular sequence of DNA in a population or gene pool (Jiang, 2013). These molecular markers are short sequences of nucleotides positioned beside the DNA sequence of the desired gene, thus, they are genetically linked and can be transferred from one generation to the next (Abu et al., 2021). The polymorphisms present in molecular markers which arise from alteration of nucleotides or mutations within the genomic loci make them useful for the identification of genetic differences between individual organisms and assessment of the relationship between breeds (Igwe, 2021). Different molecular markers have been used to assess the genetic diversity within the germplasm of orphan legumes (Table 6). The initial molecular markers were PCR-based Restriction Fragment Length Polymorphism (RFLP), Random Amplified DNA (RAPD), Amplified, Fragment Length Polymorphism (AFLP) until the development of sequencing-based molecular markers such as single nucleotide polymorphism (SNP), diversity array technology (DArT) and expressed sequence tags (ESTs) which have provided much useful information for the improvement of orphan legumes. The relative ease and reduced cost of the next-generation sequencing platforms imply that the use of SSR and/or single nucleotide polymorphisms for genetic diversity analysis and association mapping could be supplanted by genotyping by sequencing (Cullis and Kunert, 2017).

**Table 6:** Molecular markers used for genetic diversity and population studies in orphan legumes.

Plant species	Molecular markers	Utilization	References
African yam bean	Amplified fragment length polymorphisms (AFLP)	Evaluation of genetic diversity in 40–80 accessions of AYB revealed high levels of genetic diversity among the accessions with primer	Adewale et al., 2014; Ojuederie et al., 2014
	Simple sequence repeats (SSR)	Transferability of 36 SSR-derived markers from cowpea revealed considerable genetic diversity among 67 AYB accessions which could be exploited for genetic improvement.	Shitta et al., 2016
	Inter-Simple Sequence Repeat (ISSR)	Genetic variability of AYB accessions from Ebonyi State revealed a high degree of variation which could be utilized for improvement of the species	Nnamani et al., 2019
	Diversity array technology (DArT) sequencing	Genome-wide association mapping of nutritional traits of AYB using DArT Seq identified quantitative trait loci (QTL) for genes which could be useful for the improvement of the protein, oil, and starch contents of AYB	Oluwole et al., 2021b
Bambara groundnut	Amplified fragment length polymorphism (AFLP)	Profiling of the genetic diversity of 100 Bambara landraces from diverse regions of Tanzania using AFLP markers. Landraces were clustered into two groups which correlated with their geographic origin and phenotypic traits.	Ntundu et al., 2004
	Random amplified polymorphic DNA (RAPD)	Genetic diversity of Bambara groundnut accessions from Burkina Faso.	Konate et al., 2019
		Genetic diversity in landraces of Bambara groundnut found in Namibia	Mukakalisa et al., 2017
		Considerable genetic diversity relationship was found among 25 African accessions of Bambara groundnut	Amadou et al., 2001
	Random amplified polymorphic DNA (RAPD) and Inter-Simple sequence repeats (ISSR)	Assessment of the genetic diversity of 363 Bambara accessions from 5 geographical regions using 65 loci obtained from ISSR and RAPD markers. Accessions were grouped into West and East Africa populations with West Africa identified as the center of diversity of the bean, mostly cultivated in Nigeria and Cameroon	Rungnoi et al., 2012
	Microsatellites	Assessment of genetic diversity and structure of South African Bambara groundnut landraces	Kambou et al., 2021
		Identification of cultivars with a wider genetic base	Minnaar-Ontong et al., 2021
	Diversity array technology (DArT) sequencing Single nucleotide polymorphism (SNP)	Assessment of genetic diversity and population structure of Bambara groundnut landraces from different geographical regions in Africa (West, Central, Southern, and East Africa) and an unknown origin in the UK	Uba et al., 2021
Kersting’s groundnut	Diversity array technology (DArT) sequencing	Assessment of genetic diversity and population structure of 217 Kersting’s groundnut accessions from five west African countries using 886 DArTseq generated SNP markers. Despite the low polymorphism information content (0.059) the SNPs gave greater density which enhances their effectiveness in quantification of the genetic diversity and discrimination of the accessions.	Kafoutchoni et al., 2021
		Assessment of the potential effects of climate variations on suitable environments for Kersting’s groundnut cultivation, and subsequent distribution around four West-African countries using genetic information from DArTseq and ecological niche modelling. Large areas with suitable conditions for the cultivation of Kersting’s groundnut and genetic populations of the landraces were determined.	Coulibaly et al., 2022
	Single nucleotide polymorphism (SNP)	GWAS analysis revealed 10 significant marker-trait associations, of which six SNPs were consistent across environments. The genomic selection through cross-validation showed moderate to high prediction accuracies for leaflet length, seed dimension traits, 100 seed weight, days to 50% flowering, and days to maturity.	Akohoue et al., 2020; Paliwal et al., 2021
Lima bean (LB; *Phaseolus lunatus*)	Random Amplified Polymorphic DNA (RAPD)	Assessment of genetic variability of 46 accessions of the Lima bean including 16 wild forms and 30 landraces. Higher genetic diversity was observed among landraces than among wild forms.	Fofana et al., 1997
	Chloroplast DNA (cpDNA)	Two chloroplast DNA probes revealed genetic diversity among 152 accessions of LB including wild forms and landraces with a wide distribution range of two separate groups Mesoamerican and Andean.	Fofana et al., 2001
	Simple sequence repeats (SSR)	Genetic diversity, structure, and gene flow of 11 wild populations of *Phaseolus lunatus* L. in four regions of traditional agriculture in the Yucatan Peninsula, Mexico, part of the putative domestication area of its Mesoamerican gene pool were determined. Increased diversity	Martínez-Castillo et al., 2006
		The study estimated the natural outcrossing rates and genetic diversity levels of Lima bean from Brazil useful for conservation and breeding.	Penha et al., 2017; Gomes et al., 2019
	Inter-simple sequence repeats (ISSR)	The ISSR analysis revealed a wide genetic diversity among 23 LB accessions from Timor Island and grouped them into two main groups of “plain” seed group and “pattern” seed group.	Bria and Bani, 2021
Jack bean (JB)	Sequence-related amplified polymorphism (SRAP)	Genetic diversity and relationship among 29 accessions of JB from 16 countries revealed a low variation of five cluster groups composed of different accessions with different phenotypic traits.	Liu et al., 2014

The identification of quantitative trait loci (QTLs) for several traits in orphan legumes and the development of codominant molecular markers and linkage maps for some orphan crops have been made possible through marker-assisted selection with specific molecular markers. There is an urgent need to link phenotypic data with the genotypic data to appropriately select the landraces of these orphan legumes for genetic improvement.

Using a high-throughput DArTseq genotype-by-sequencing SNP approach, Oluwole et al. (2021b) generated a total of 3.6 k SNPs out of which 2.48 K quality SNPs were used for Genome-Wide Association Study (GWAS) in the AYB population. They identified quantitative trait loci (QTL) for genes that could be useful for the improvement of the protein, oil, and starch contents of AYB. Likewise, 493 SNPs were used for the genotyping of a population of 281 Kersting’s groundnut accessions from Benin using the DArTseqTM approach with about 10.6% of the SNPs found to be aligned to the reference genomes of adzuki bean and mung bean, an indication of an evolutionary relationship of Kersting’s groundnut with adzuki bean and mung bean (Akohoue et al., 2020; Paliwal et al., 2021). Uba et al. (2021) utilized DArTseq and SNP for genetic diversity and population structure studies on some genotypes of BG obtained from Nigeria, Cameroun, West Africa, East, and Southern Africa regions. The analyses of the results revealed that the mean gene diversity was highest 0.478) in Nigeria/Cameroon and West Africa region within the populations across the five regions and revealed the highest Shannon diversity index (0.787) in the West African region. Likewise, it also indicated that among the populations the genotypes in the unknown origin population from the United Kingdom was more closely related to the Western Africa population (0.018), and then the Nigeria/Cameroon populations (0.020) which were all grouped into a single subpopulation which was the largest based on the population structure generated by ADMIXTURE model among the 270 BG genotypes evaluated (Uba et al., 2021). Not much effort has been done in the use of gene technology or modern biotechnology for the genetic improvement of orphan legumes. Though there is public skepticism of the use of modern biotechnology for crop improvement adhering to biosafety measures and guidelines the technology could be very useful for the genetic enhancement of orphan legumes.

Advances in next-generation sequencing have provided a way for a new generation of different omics such as genomics, proteomics, metabolomics, and transcriptomics, which can proffer imperative solutions to these underutilized legumes, enhance crop improvement, and broaden the scope of the various germplasms (Popoola et al., 2020). These multi-omics approaches have been effectively utilized in elucidating growth, senescence, yield, and the responses to environmental stresses in several crops (Yang et al., 2021). Utilization of omics technologies coupled with high-throughput next-generation sequencing platforms, and bioinformatics, have allowed a greater comprehension of the plant system biology as well as the expression of genes and the various metabolic systems in plants (Ojuederie et al., 2022). The ever-increasing decline in the cost of sequencing has initiated the contribution of genomics to the improvement of orphan legumes. However, the use of omics in the characterization of orphan legumes is still in its infancy stage. This is because most of the genomes of these legumes are yet to be sequenced. Nevertheless, efforts are ongoing in the whole genome sequencing of some orphan legumes led by several organizations such as the African Orphan Crops Initiative^2^ to close the gap yet to be filled by the use of genomics transcriptomics and proteomics to extract useful information for the improvement of orphan legumes.

The sequenced genomes of legumes and the model species such as *Glycine max*, *Vigna unguiculata*, and *Medicago trunculata*, would speed up genomic advancements through comparative genomics in other important orphan legumes such as cluster bean, Dolichos bean AYB, as well as winged bean, which are still being sequenced (Dhaliwal et al., 2020).

### Potential of Gene Editing Tools in Orphan Legumes

Gene editing entails the use of engineered nucleases to instigate cellular DNA repair pathways to make precise, site-directed alterations to an organism’s genome (Bhowmik et al., 2021). Over the years, several genome editing techniques have been developed, including Zinc-finger nucleases (ZFNs), meganucleases, transcription activator-like effector nucleases (TALENs), clustered regularly interspaced short palindromic repeats (CRISPR)-CRISPR-associated protein 9 (Cas9) and homing endonucleases (Gaj et al., 2016). However, CRISPR/Cas9-mediated gene editing has emerged as the most straightforward, adaptable, and precise strategy for genetic manipulation in plants (Bhowmik et al., 2021; Nadakuduti and Enciso-Rodríguez, 2021). The mechanism of action that underpins the CRISPR technology has been extensively reviewed elsewhere (Gaj et al., 2016; Li et al., 2020; Wada et al., 2020; Bhowmik et al., 2021).

While the recalcitrant nature of legumes to *in vitro* gene transfer, low amenability to transformation, and regeneration efficiencies have posed a significant hurdle to the application of the CRISPR technology, some level of success has been recorded. CRISPR/Cas9-assisted gene editing has been achieved in some legume crops, including *Medicago truncatula* and *Lotus japonicus*, cowpea, and soybean (Wang et al., 2017; Zheng et al., 2020; Bhowmik et al., 2021). The success of the technique in these crops shows promise for its cross-application in underexploited legumes (Bhowmik et al., 2021). A critical requirement for improving crops by this technique is in-depth bioinformatic and genomic information, as the technology relies on functionally characterized target genes (Wolter et al., 2019). Primarily, the phytoene desaturase (PDS) gene which encodes an enzyme involved in carotenoid biosynthesis is the most commonly targeted gene in orphan legumes (Badejo, 2018; Venezia and Creasey Krainer, 2021). Furthermore, researchers at the international crops research institute for the semi-arid tropics (ICRISAT) are currently experimenting with the technique to knock down flowering time and photoreceptor genes correlated with photoperiod sensitivity in pigeons pea (ICRISAT, 2020).^3^ Other traits in orphan legumes that can be targeted for improvement using the CRISPR/Cas system include disease resistance, salinity tolerance, biomass yield, grain yield and quality, and nutrient use efficiency (Rasheed et al., 2021).

Albeit CRISPR provides an unparalleled opportunity to improve traits in the underutilized legumes, the first step remains to establish an efficient transformation protocol for guide RNA (gRNA) delivery and the availability of complete genome sequences in public databases. These are both lacking for the orphan legumes which this review focuses on Mousavi-Derazmahalleh et al. (2019). While the Bioscience eastern and central Africa International Livestock Research Institute (BecA-ILRI) has achieved significant progress in sequencing the entire genome of AYB, the availability of whole-genome sequences and annotations of the underexploited legumes could accelerate the deployment of CRISPR/Cas9 technology to enrich their commercial value, and improve traits associated with photosensitivity, prolonged maturity period, and hard seed coat responsible for long cooking times (ACACIA Africa, n.d.).^4^

Despite the potential benefits of CRISPR technology to underexploited legumes, regulatory bottlenecks remain a significant matter of concern (Bhowmik et al., 2021). Recently, a ruling by the European Court of Justice declared that targeted mutagenesis *via* genome editing should be subject to rigorous GMO regulations, even though the product is free of any foreign gene (Wolter et al., 2019; Zaidi et al., 2020). This calls for concerted efforts from policy makers and scientists to design a comprehensive framework for CRISPR-generated crops integration. In addition, large-scale field trials are also required to evaluate the performance of CRISPR-generated crops for traits that may be compromised owing to the disruption of specific genes (Zaidi et al., 2020).

### Speed Breeding in Orphan Legumes

Speed breeding is revolutionizing plant breeding, making it possible to have several generations of crops within a year. It utilizes enhanced light-emitting diode (LED) supplemental lighting and day-long regimes of up to 22 h at temperatures maintained between 17°C and 22°C to enhance photosynthesis and early flowering of the plants, which results in rapid growth and better yield. During the process, the LED lighting increases the breeding cycle of the exposed plants. Scientists at the University of Queensland, Australia, developed this technique. They successfully used it to increase the growth of spring wheat (*Triticum aestivum*), durum wheat (*T. durum*), barley (*Hordeum vulgare*), Chickpea (*Cicer arietinum*) per year in a temperature-controlled glasshouse fitted with high-pressure sodium lamps for up to six generations and four generations for canola (*Brassica napus*), in contrast to 2–3 generations under normal glasshouse conditions or field using conventional breeding methods (Watson et al., 2018). They achieved a significant reduction in the time taken for anthesis to occur for all crop species at 22 h photoperiod compared with the 12-h day-neutral photoperiod conditions, with a mean decrease of 22 ± 2 days (wheat), 64 ± 8 days (barley), 73 ± 9 days (canola) and for Chickpea, 33 ± 2 days (Watson et al., 2018). It, therefore, implies that plant breeders would be able to accelerate the rate of genetic improvement of several crops such as wheat, barley, rape, and pea for increased yield, disease resistance, and climate resilience in crops. The neglected and underutilized legumes could also benefit from this modern plant breeding technique, especially as most climate-resilient crops possess a rich nutritional profile.

Gaur et al. (2007) and O’Connor et al. (2013) successfully developed protocols for speed breeding of chickpea (Gaur et al., 2007; Chiurugwi et al., 2018) as well as *Arachis hypogea*, which resulted in a reduction in the generation time to 89 days from the usual 145 days in the second filial generation hybrids (O’Connor et al., 2013; Chiurugwi et al., 2018). They employed controlled temperature and constant light to fast-track plant development and accelerated a single seed descent (SSD) breeding program. Gaur et al. (2007) was able to achieve three seed-to-seed generations within a year by carrying out two generations under open field conditions and one under rainout shelters by exposing plants to lengthened (24-h) photoperiod (Saxena et al., 2019). Protocols have also been developed to shorten the generation time in some orphan legumes and oilseeds. These include lupin (*Lupinus* sp.; Croser et al., 2016), chickpea (*Cicer arietinum*; Sethi et al., 1981; Watson et al., 2018), subterranean clover (*Trifolium subterraneum*; Pazos-Navarro et al., 2017), lentil (*Lens culinaris*) as well as broad beans (*Vicia faba*; Mobini et al., 2015; Lulsdorf and Banniza, 2018). The application of cytokinins and auxins to induce early flowering enabled Mobini et al. (2015) to increase the number of generations to 7 generations for Faba bean (*Vicia faba* L.) and eight generations for lentil (*Lens culinaris* Medik), after which immature seeds were harvested for generation advancement (Saxena et al., 2019).

The AYB (*Sphenostylis stenocarpa*) has recently received more attention as a potential food and nutrition security crop. However, it has a long generation time between 6 and 7 months hence it is grown once a year. Could this dual-purpose crop that produces edible seeds and underground tubers also benefit from speed breeding? Some landraces of AYB are tuber producing while others are not. This characteristic has been linked to the photoperiod sensitivity of the crop, with short photoperiods being suggested as a probable stimulant for tuberization in the species. Using the speed breeding approach, which requires more extended photoperiod of 22 h to shorten the breeding cycle, may not initiate tubers in tuber-producing landraces but may shorten the generation time of the crop. However, a major challenge to the use of speed breeding techniques in Africa is the availability of a constant power supply. In some countries utilizing the technology, containers used for conveying manufactured goods across borders are being converted to a speed breeding facility with LED lights installed and powered by solar panels. Alternatively, a solar-powered glasshouse with LED fitted lighting could be used for speed breeding and adapted for orphan or underutilized legumes in Africa by the National Agricultural Research Centers in the various countries where these legumes are cultivated.

With the speed breeding technique, plant breeders and plant molecular geneticists would hasten the genetic variation inherent in wild relatives of these orphan legumes, thereby introducing elite varieties that will be widely accepted and grown by farmers. The ongoing discoveries of the vast genetic resources and nutraceutical components of these lesser-known legumes are increasingly attracting research and funding attention as potential future crops for sustainable agriculture by CGIAR, IITA, and other Genebank institutes around the world. International agricultural organizations such as the West Africa Centre for Crop Improvement (Ghana), the BeCa-ILRI Hub (Kenya), the World Vegetable Center (Taiwan), the African Orphan Crops Consortium (Kenya), Crops for the Future (Malaysia), the Global Pulse Confederation (UAE) and the CGIAR Centres and Research Programmes like the International Crops Research Institute for the Semi-Arid Tropics (ICRISAT), the International Institute of Tropical Agriculture (IITA), and the International Center for Agricultural Research in the Dry Areas are well-positioned to establish speed breeding facilities to give a boost to the breeding programs of these orphan legumes for enhanced utilization and food and nutrition security. Despite the potential of speed breeding in reducing the breeding cycle in crops, it has some limitations. The success of speed breeding depends on the crop and species and proper regulation of environmental factors such as the photoperiod, temperature, light intensity, and relative humidity. It may also be challenging to utilize speed breeding for short-day plants that require less light for floral induction. Simulating breeding set-ups that combine rapid generation advances with other technologies like genomic selection (GS) would be an excellent approach to optimize breeding methods cost-effectively (Hickey et al., 2019). Genomic selection (GS) accelerates the selection accuracy of superior genotypes, germplasm enhancement and aids in selecting targeted genes for particular traits from Genebank accession to elite lines. Thus, the genetic gain of key traits can be accelerated using integrative approaches of speed breeding and genomic selection, permitting more rapid production of improved cultivars.

## Conclusion

The need to introduce these orphan legume crops into the food systems in Africa and other poverty-prone countries to attain zero hunger, improved nutrition, and sustainable agriculture goals of the SDG by 2030 is nonnegotiable. Their dense network of cheap, high, and quality protein, dietary fiber, vitamins, minerals, carbohydrates, low fat, the presence of diverse nutraceuticals and pharmaceutical components that can help prevent or alleviate diet-mediated disease cannot be overemphasized. Without any doubt, the inclusion of underutilized legumes into the dietary cycle could offer long-lasting solution to the high prevalence of malnutrition, hunger, and protein deficiencies in SSA malnutrition, hunger, and protein deficiency, will be a long-lasting solution due to its dense nutrient content, good quality protein, and micronutrients. Currently, the potentials of these minor legumes have begun to receive scientific attention. The target is to improve crop yield, eradicate toxic ANTs, reduce the cooking time of the seeds, speed up maturity rate, gain consumer acceptance, and solve food and protein insecurity. Efforts to elevate these nutrient-rich crops to the top of the food basket of any nation are demanding. However, modern technologies such as speed breeding and CRISPR/Cas9 in an integrative approach with genomic selection and other high-throughput methods could fast track the breeding cycle of orphan legumes to develop new varieties with traits that enable more than one cycle of cultivation per year. Still, with the various efforts being made worldwide, it is hoped that these legumes will contribute immensely to the food and nutritional security of most households and may likely become staple crops for Africans in the not-so-distant future.

## Author Contributions

JOP – conceived the idea, wrote the first draft, search the literature, and reviewed the manuscript. OSA and OBO – contributed to the writing and review of the manuscript. BDA – contributed to the writing and review. OCA – literature search and contributed to the writing. DIE – literature search. OAO – contributed to the writing of the manuscript. TTA – contributed to the writing. OOO – reviewed and approved the final manuscript. All authors contributed to the article and approved the submitted version.

## Conflict of Interest

The authors declare that the research was conducted in the absence of any commercial or financial relationships that could be construed as a potential conflict of interest.

## Publisher’s Note

All claims expressed in this article are solely those of the authors and do not necessarily represent those of their affiliated organizations, or those of the publisher, the editors and the reviewers. Any product that may be evaluated in this article, or claim that may be made by its manufacturer, is not guaranteed or endorsed by the publisher.

## Footnotes

^1^ https://www.tridge.com/intelligences/butter-bean/export

^2^ http://africanorphancrops.org

^3^ https://www.icrisat.org/cgiar-focus-on-gene-editing-crops-for-a-food-and-income-secure-future/

^4^ https://acaciaafrica.org/bioinformatics-community-practice/full-genome-sequencing-and-annotation-of-the-african-yam-bean/
